# Late gestational nutrient restriction in primiparous beef females: nutrient partitioning among the dam, fetus, and colostrum during gestation

**DOI:** 10.1093/jas/skad195

**Published:** 2023-06-14

**Authors:** Colby A Redifer, Lindsey G Wichman, Abigail R Rathert-Williams, Harvey C Freetly, Allison M Meyer

**Affiliations:** Division of Animal Sciences, University of Missouri, Columbia, MO 65211, USA; Division of Animal Sciences, University of Missouri, Columbia, MO 65211, USA; Division of Animal Sciences, University of Missouri, Columbia, MO 65211, USA; USDA, ARS, Roman L Hruska US Meat Animal Research Center, Clay Center, NE 68933, USA; Division of Animal Sciences, University of Missouri, Columbia, MO 65211, USA

**Keywords:** beef heifer, developmental programming, fetal growth, metabolites, pregnancy

## Abstract

Fall-calving primiparous crossbred beef females [body weight (BW): 451 ± 28 (SD) kg; body condition score (BCS): 5.4 ± 0.7] were allocated by fetal sex and expected calving date to receive either 100% (control; CON; *n* = 13) or 70% (nutrient restricted; NR; *n* = 13) of metabolizable energy and metabolizable protein requirements for maintenance, pregnancy, and growth from day 160 of gestation to calving. Heifers were individually-fed chopped poor quality hay and supplemented to meet targeted nutritional planes based on estimated hay intakes. Dam BW, BCS, backfat, and metabolic status were determined pre-treatment, every 21 d (BW and metabolic status) or 42 d (BCS and backfat) during gestation, and post-calving. At birth, calf BW and size were measured, and total colostrum from the most full rear quarter was collected pre-suckling. Data were analyzed with nutritional plane, treatment initiation date, and calf sex (when *P *< 0.25) as fixed effects. Gestational metabolites included day and nutritional plane × day as repeated measures. During late gestation, CON dams gained (*P *< 0.01) maternal (non-gravid) BW and maintained (*P *≥ 0.17) BCS and backfat, while NR dams lost (*P *< 0.01) maternal BW, BCS, and backfat. Circulating glucose, urea N, and triglycerides were less (*P *≤ 0.05) in NR dams than CON at most late gestational timepoints after treatment initiation. Circulating non-esterified fatty acids were greater (*P *< 0.01) in NR dams than CON. Post-calving, NR dams weighed 63.6 kg less (*P *< 0.01) and were 2.0 BCS less (*P *< 0.01) than CON. At 1 h post-calving, NR dams had less (*P *= 0.01) plasma glucose and tended to have less (*P *= 0.08) plasma triglycerides than CON. Nutrient restriction did not affect (*P *≥ 0.27) gestation length, calf birth weight, or calf size at birth. Colostrum yield was 40% less (*P *= 0.04) in NR dams than CON. Protein and immunoglobulin concentrations were greater (*P *≤ 0.04), but free glucose and urea N concentrations were less (*P *≤ 0.03), in colostrum of NR dams than CON. Colostrum total lactose, free glucose, and urea N were less (*P *≤ 0.03) in NR dams than CON, but total protein, triglycerides, and immunoglobulins were not affected (*P *≥ 0.55). In summary, beef heifers experiencing late gestational nutrient restriction prioritized partitioning nutrients to fetal growth and colostrum production over maternal growth. During undernutrition, fetal and colostral nutrient demands were largely compensated for by catabolism of maternal tissue stores.

## Teaser

Beef heifers experiencing late gestational nutrient restriction prioritized partitioning nutrients to fetal growth and colostrum production over maternal body tissues. Nutrient restricted females lost body weight and body condition during late gestation, but calf size at birth and colostrum total nutrients and immunoglobulins, except lactose, did not differ from adequately-fed females.

## Introduction

Maternal energy and protein requirements increase in the late gestation beef female to support exponential growth of the uteroplacenta, fetus, and mammary gland (Ferrell et al., 1976; NASEM, 2016). The first-parity female has the greatest challenge, as maternal growth is still occurring, and she is establishing a successful gravid uterus and mammary gland for the first time. If nutrients are consumed at or above requirements, then physiological productivity above maintenance can theoretically be maximized. Unfortunately, late gestation beef females are often faced with limited nutrient availability due to reduced quality of mature or dormant forage or limited forage quantity in the dormant season or drought conditions (DelCurto et al., 2000; Caton and Hess, 2010). When undernutrition occurs, there is competition for nutrients to be partitioned across physiological functions in the female; however, the priority of these is not absolute or well understood (Short and Adams, 1988).

Previous gestational nutrient restriction research has generally associated decreased nutrient delivery to the dam with programming fetal growth and development, resulting in poor postnatal outcomes (Wu et al., 2006; Reynolds et al., 2010). Nutrients partitioned to the mammary gland during pregnancy for colostrum production are critical, but the effects of maternal nutrition are poorly understood (McGee and Earley, 2019). While gestational undernutrition has had detrimental effects on the dam, fetus, and colostrum separately, there is the critical need to conduct an intensive study that evaluates these three nutrient sinks simultaneously in the first-parity beef female.

A large experiment investigated the impacts of late gestational nutrient restriction in individually-fed first-parity beef females on prenatal and postnatal nutrient availability and utilization by the offspring. We hypothesized that late gestational nutrient restriction decreases circulating post-absorptive nutrient concentrations, causes mobilization of maternal body reserves, and ultimately reduces uteroplacental and mammary gland nutrient delivery, negatively affecting fetal growth and colostrum production. The specific objectives of the current paper were to investigate the effects of late gestational maternal nutrient restriction on 1) maternal body weight (**BW**), body condition, and circulating metabolites during gestation, 2) calf size at birth and calving difficulty, and 3) colostrum yield and composition.

## Materials and Methods

The University of Missouri Animal Care and Use Committee approved animal care and use in this study (Protocol #9877), which took place at the University of Missouri Beef Research and Teaching Farm (Columbia, MO).

### Animal management and diets

Single-sired fall-calving Hereford × Simmental-Angus crossbred beef heifers [437 ± 5 d (SD throughout methods) of age] were bred to a single Angus sire via a fixed-time artificial insemination protocol on November 23, 2019. Heifers were then fitted with heat detection patches and monitored daily over the next 3 mo for return to estrus and were artificially inseminated to the same sire to obtain sufficient numbers. Via transrectal ultrasonography, pregnancy was confirmed approximately 35 d after artificial insemination and fetal sex was determined between days 60 and 80 of gestation. From artificial insemination through mid-gestation, heifers grazed stockpiled tall fescue then were moved to a drylot with ad libitum access to tall fescue-based hay. During this time, heifers were supplemented with whole corn, dried distillers grains with solubles (**DDGS)**, and soyhull pellets to meet or exceed nutrient requirements and maintain a target body condition score (**BCS**; 1 to 9 scale, 1 = emaciated, 9 = obese; Wagner et al., 1988) of 5 to 6. Twenty-six pregnant heifers (initial BW = 451 ± 28 kg, initial BCS = 5.4 ± 0.7) were allocated by BW, BCS, fetal sex, and expected calving date to 1 of 2 late gestational nutritional planes from day 160 of gestation to parturition. Control (**CON**; *n* = 13) heifers were individually-fed 100% of metabolizable energy (**ME**) and metabolizable protein (**MP**) requirements for maintenance, pregnancy, and growth, whereas nutrient restricted (**NR**; *n* = 13) heifers were individually-fed 70% of ME and MP requirements.

On approximately day 139 of gestation, pregnant heifers were moved to partially-covered pens (3.7 m × 15.8 m) equipped with a Calan gate feeding system (Calan Broadbent Feeding System, American Calan, Northwood, NH) for acclimation to individual feeding in the electronic gate system and transition from tall fescue hay to the chopped sorghum sudan hay fed during the treatment period. During the acclimation period, all animals were offered ad libitum chopped hay and received similar amounts of DDGS and soyhull pellets. At treatment initiation, heifers were allocated to 1 of 12 pens, penned by nutritional plane (*n* = 2 to 3 heifers per pen).

From day 160 of gestation to parturition, nutrient requirements were using an expected calf birth weight of 34 kg and projected maternal average daily gain of 0.36 kg/d. Metabolizable energy for maintenance (**ME**_**m**_) was based on data for heifers in confinement (Freetly and Hales, personal communication). An equation for ME for conceptus (**ME_y_**) was used as published previously (Freetly et al., 2005). Equations from NASEM (2016) were utilized for ME for gain (**ME_g_**); MP for maintenance (**MP_m_**; Eq. 11–17), conceptus (**MP_y_**; Eq. 13–43 and 13–44), and gain (**MP_g_**); and gravid uterus weight (Eq. 13–27). Requirements were calculated from the following equations; total ME (ME_m_, ME_y_, and ME_g_) and MP (MP_m_, MP_y_, and MP_g_) were then summed.


$${\text{ME}}_{\text{m}}(Mcal/d)=0.138{\left(heifer\;BW\right)}^{0.75}$$

where heifer BW (kg) = BW − gravid uterus weight;


$${\text{ME}}_{\text{y}}\text{(Mcal/d) = gravid uterus weight (kg)}=\text{CBW(0}{\text{.01828e}}^{\text{(0.02}-\text{0.0000143t)t}}\text{);}$$

CBW (kg) = calf birth weight;

t = day of gestation


$${\text{ME}}_{\text{y}}\text{(Mcal/d)}=\text{(CBW(0.4504}-\text{0}{\text{.000766t)e}}^{\text{(0.03233}-\text{0.0000275t)t)}}\text{)/ 1, 000}$$

where CBW (kg) = calf birth weight;

t = day of gestation


$${\text{ME}}_{\text{g}}\text{(Mcal/d)}=\text{4.9}$$

$${\text{MP}}_{\text{m}}\text{ (g/d) = 3}{\text{.8(shrunk heifer BW)}}^{\text{0.75}}$$

where shrunk heifer BW (kg) = 0.96(BW − gravid uterus weight);


$$\text{gravid uterus weight (kg)}=\text{CBW(0}{\text{.01828e}}^{\text{(0.02}-\text{0.0000143t)t}}\text{);}$$

CBW (kg) = calf birth weight;

t = day of gestation


$${\text{MP}}_{\text{y}}\text{(g/d)}=\text{Ypn/ 0.65}$$

where $Ypn(netproteinretainedasconceptus;g/d)=CBW((0.001669--0.00000211t)e(0.0278--0.0000176t)t)6.25;\text{Ypn (net protein retained as conceptus;}\;\text{g/d) = CBW((0}\text{.001669 -- 0}\text{.00000211t)}{\text{e}}^{(\text{0}\text{.0278 -- 0}\text{.0000176t})\text{t}}\text{)6}\text{.25;}$$Ypn(netproteinretainedasconceptus;g/d)=CBW((0.001669--0.00000211t)e(0.0278--0.0000176t)t)6.25;\text{Ypn (net protein retained as conceptus;}\;\text{g/d) = CBW((0}\text{.001669 -- 0}\text{.00000211t)}{\text{e}}^{(\text{0}\text{.0278 -- 0}\text{.0000176t})\text{t}}\text{)6}\text{.25;}$

CBW (kg) = calf birth weight;

t = day of gestation


$${\text{MP}}_{\text{g}}\text{(g/d)}=121.46$$

Metabolizable protein requirements were converted to crude protein (**CP**) requirements using the equation CP (g/d) = MP/ 0.64, based on 80% true protein × 80% digestibility (NASEM, 2016). Requirements for ME and CP were adjusted weekly based on the most recent dam BW (recorded every 21 d) and day of gestation (the first day of that week). Requirements were then multiplied by a factor of 1.0 or 0.7 to obtain final ME and CP targets for each individual CON and NR heifer, respectively. Control heifers were expected to maintain a BCS of 5 to 6 and gain maternal (non-gravid) BW reaching 80% of their estimated mature BW (590 kg) by parturition.

From days 160 to 265 of gestation, diets were based on chopped sorghum sudan hay [1.74 Mcal ME/kg, 6.69% CP, 72.0% neutral detergent fiber (**NDF**), 52.8% acid detergent fiber (**ADF**); dry matter (**DM**) basis]. Sorghum sudan hay was offered at 1.56% BW on DM basis. Feeding poor quality forage allowed animals fed both nutritional planes to consume ad libitum hay, preventing any pica in the NR dams without exceeding the ME and CP targets at any point. This also ensured that physical gut fill, at least from a forage standpoint, was as similar as possible between nutritional planes during nutrient restriction.

Beginning on day 266 of gestation, heifers were transitioned (over 3 d) to chopped endophyte-infected tall fescue-based hay (1.90 Mcal ME/kg, 7.22% CP, 65.1% NDF, 43.2% ADF; DM basis) fed through parturition. This allowed for less supplement to meet ME and CP targets during late gestation and lactation. Tall fescue hay was offered at 1.87% BW on DM basis.

Based on expected individual hay intakes, heifers were supplemented daily with whole corn (3.18 Mcal ME/kg, 8.44% CP; DM basis), DDGS (3.22 Mcal ME/kg, 31.0% CP; DM basis), and soyhull pellets (2.89 Mcal ME/kg, 11.2% CP; DM basis) to meet their ME and CP targets. Supplement for each heifer was reformulated weekly to meet ME and CP targets, and then supplement for each day was weighed individually. Hay was offered at a similar % of BW, and the NR nutritional plane was achieved solely by a reduction in supplement provided, resulting in the decrease in energy and protein. The corn:soyhull and DDGS:soyhull ratios remained similar for animals within a nutritional plane but varied between nutritional planes as necessary to remain between 98% and 104% of each heifer’s ME and CP targets.

Nutrient analysis of the hay and supplement feedstuffs was conducted throughout the experiment to reformulate the daily supplement to meet ME and CP targets. Before grinding and feeding, core samples of sorghum sudan and tall fescue hay round bales representing every bale in a grinding group were submitted to Cumberland Valley Analytical Services (CVAS, Inc., Waynesboro, PA) for wet chemistry analysis of DM, CP (AOAC, 990.03), ADF (AOAC, 973.18), and NDF (Van Soest et al., 1991). Acid detergent fiber values were used to calculate total digestible nutrients (**TDN**). For sorghum sudan hay, TDN (%) = 88.9 − (0.779 × % ADF) from NFTA (1993). For tall-fescue based hay, TDN (%) = 98.625 − (1.048 × % ADF) from Holman et al. (2021). Metabolizable energy was determined using the equation ME (Mcal/kg DM) = % TDN × 0.0362. This is based on 4.409 Mcal digestible energy/kg of TDN × 0.82 digestible energy:ME ratio (NASEM, 2016). Samples of whole corn, DDGS, and soyhull pellets were also submitted to CVAS for wet chemistry analysis of DM (AOAC, 930.15) and CP (AOAC, 990.03) before feeding each new load of a feedstuff. Values of ME from NASEM (2016) for DDGS and NRC (2000) for whole corn and soyhull pellets were used in supplement formulation.

Supplement was fed every morning at approximately 0700 hours in a feed pan to prevent wastage and was consumed before morning delivery of hay. Supplement refusals were not left by any female during gestational treatments. Hay was delivered in two equal portions to each individual heifer every morning (0730 hours) and evening (1900 hours). Heifers had ad libitum access to water and a trace mineralized salt block (96% NaCl min., 99% NaCl max., 2,400 mg/kg Mn min., 2,400 mg/kg Fe min., 260 mg/kg Cu min., 380 mg/kg Cu max., 320 mg/kg Zn min., 70 mg/kg I min., 40 mg/kg Co min., Big 6 Mineral Salt, Compass Minerals America Inc., Overland Park, KS). Approximately once monthly, pen floors were scraped clean and rebedded with fresh sawdust.

### Nutrient intake calculations

While animals were being individually-fed in Calan gates, representative subsamples of the chopped hay, supplement feedstuffs, and hay refusals were collected and analyzed to calculate nutrient intakes. Chopped hay samples were collected at each feeding and composited by week, and hay refusals were weighed back twice weekly, before the morning feeding, and were sampled for each animal individually (composited by week) for DM analysis. Individual hay DM intake (**DMI**) was calculated weekly using actual hay and refusal DM data. Whole corn, DDGS, or soyhull pellet samples were taken daily and composited by month for CP analysis by CVAS using wet chemistry. Beginning on day 160 of gestation, DM, ME, and CP intakes from each ingredient were calculated and summed by day, then averaged by week. Intakes for the week beginning on day 272 of gestation consist of the average intake from that day until the day of calving (average: day 279; range: days 274 to 286). Hay intakes were not recorded for 1 CON heifer from days 160 to 176 of gestation and 1 NR heifer from days 226 to 232 of gestation due to veterinary care altering how hay could be delivered (unrelated to treatment).

### Gestational data collection

Consecutive 2-d BW and jugular blood samples were collected before nutritional plane treatment allocation (days 158 and 159 of gestation). Additional gestational BW and jugular blood samples were collected at 21-d intervals (2-d BW every 42 d and on day 265 of gestation) occurring on days 181, 202, 223, 244, and 265 of gestation. These occurred immediately before the morning delivery of supplement and hay and within ± 1 d of the actual day of gestation. Dam BCS was assessed by the same two trained technicians on days 158, 202, 244, and 265 of gestation, and scores were averaged. Backfat thickness between the 12th and 13th ribs was measured by a trained technician on days 158, 202, and 244 of gestation using an Aloka 500-SSV ultrasound machine (Aloka Co. Ltd., Tokyo, Japan) with an Aloka 17 cm 3.5 MHz linear transducer (UST-5044-3.5) and standoff. Two separate images were measured, and backfat thickness was determined at a point three-quarters the length of the longissimus muscle from the spine.

### Calving data and sample collection

During the peripartum period, under roof and outdoor lighting allowed for continuous monitoring of females. Beginning on day 274 of gestation, heifers were closely monitored 24 h per day by trained personnel to detect when heifers were in stage II of parturition. Once stage II was detected, the heifer was continuously monitored. Calving assistance was provided if there was a prolonged duration since first appearance of fetal membranes, if progress slowed during contractions, or if it was evident the calf was presenting abnormally. The average calving date was September 16 ± 32.9 d, and there was no perinatal calf death loss.

#### Calf size at birth

Following the successful first standing of a calf but before suckling, calves were removed from the dam, processed, and calf size at birth was determined (0.9 ± 0.3 h of age). Each calf had the sex recorded and was given an ear tag for visual identification. Calf birth weight was measured using an electronic hanging scale with a calf sling placed under the abdomen of the calf. Using a flexible measuring tape, calf body size measures were recorded. Heart girth was measured as the body circumference immediately behind the shoulders and front legs, perpendicular to the spine. Abdominal girth was measured by placing the tape measure around the abdomen over the umbilicus, perpendicular to the spine. Flank girth was measured as the body circumference immediately in front of the hooks, perpendicular to the spine. Shoulder to rump length was measured along the spine from the front of the shoulder blades to the end of the tailhead. Cannon bone circumference was measured at the smallest circumference of a rear cannon bone. Cannon bone length was measured from the center of the knee to between the declaws along the back of a calf’s front cannon bone. Coronet circumference was measured at the hairline between hoof and hair of a front leg. Height at the shoulder was recorded as height at the dorsal edge of the shoulder, with the head up and feet set square, using an aluminum height measuring stick. As an indicator of calf shape, calf ponderal index was calculated using the equation ponderal index = calf birth weight (kg)/shoulder to rump length (m)^3^. Calf heart girth:length ratio (both in cm) was also calculated. Finally, calf volume was calculated using the equation volume (L) = [π × average girth radius (cm)^2^ × shoulder to rump length (cm)]/ 1,000.

Calf longissimus muscle area between the 12th and 13th ribs was measured by a trained technician on day 7 of age using an Aloka 500-SSV ultrasound machine and 12.5 cm 3.5 MHz linear transducer (UST-5044-3.5; typically used for swine carcass ultrasound) and standoff. Duplicate images were saved onto an external memory source, and the longissimus muscle cross-section was traced using ImageJ software on a computer. Each image was traced on two separate occasions, by the same technician, resulting in four measurements averaged for analysis.

#### Colostrum collection

With the calf separated from the dam, dams were moved into a chute for colostrum collection (0.9 ± 0.3 h postpartum). Calves were continuously monitored from the time of parturition until colostrum collection to ensure suckling did not occur before sampling. The most full rear quarter was selected based upon visual inspection and palpation. The udder and teat were cleaned using infant care wipes to remove manure and dirt. The single quarter was completely hand-milked without exogenous oxytocin administration. Following colostrum collection, the dam and calf were returned to their pen to allow the calf to nurse the remaining three quarters and be naturally reared by its dam. Single-quarter colostrum volume and weight were recorded. We have previously demonstrated that the single most full rear quarter yield is a strong predictor of total colostrum yield in beef cattle (r^2^ = 0.85; Rathert-Williams et al., 2023). Sample aliquots were frozen in 2-mL microcentrifuge tubes at −20°C for later analysis.

#### Maternal performance post-calving

Before colostrum collection, a 1 h post-calving jugular blood sample was obtained from each dam. Post-calving 2-d BW were collected on days 1 and 2 of lactation between 1500 and 1800 hours. If calving occurred before 0900 hours, then the day of calving was considered day 1 of lactation; if calving occurred after 0900 hours, then the following day was day 1 of lactation. In the first week postpartum, maternal BCS (3.4 ± 1.1 d postpartum) and backfat thickness (1.8 ± 1.2 d postpartum) were collected as described for gestational timepoints. Average daily gain (gravid), maternal average daily gain (non-gravid), BCS change, and backfat thickness change from treatment initiation to pre-calving (gravid only) and post-calving were calculated. Maternal average daily gain was determined by subtracting the non-gravid BW at treatment initiation from the post-calving BW. The gravid uterine weight at treatment initiation was calculated using the equation described above from NASEM (2016) and the known calf birth BW of a respective dam, which was used to calculate non-gravid BW.

### Circulating metabolite analyses

Blood samples were collected into four collection tubes [two Vacutainer serum collection tubes containing no additives (Becton Dickinson, Franklin Lakes, NJ), one Monoject plasma collection tube containing 0.10 mL of 15% K_3_EDTA (Covidien, Mansfield, MA), and one Vacutainer plasma collection tube containing 15 mg of sodium fluoride and 12 mg of potassium oxalate (Becton Dickinson) for glucose determination]. Following collection, all tubes were inverted several times. Plasma tubes were immediately placed on ice, while serum tubes were allowed to clot before being placed on ice. Between 1 and 8 h after collection, all samples were centrifuged at 1,500 × *g* at 4°C for 30 min. Plasma and serum were transferred into multiple 2-mL microcentrifuge tubes and stored at −20°C until analysis.

Maternal plasma glucose, serum urea N, and serum non-esterified fatty acids (**NEFA**) were analyzed as described in Niederecker et al. (2018), and plasma triglycerides were analyzed as described in Larson-Peine et al. (2022). For each assay, samples were analyzed in duplicate, and pooled control samples were used. The intraassay and interassay CV were 2.7% and 2.2% for plasma glucose, 3.2% and 3.7% for serum urea N, 3.4% and 5.1% for serum NEFA, and 2.8% and 2.3% for plasma triglycerides, respectively.

### Colostrum nutrient analysis

Colostrum was analyzed for protein, triglycerides, lactose, free glucose, and urea N as described by Rathert-Williams et al. (2023). All samples were run in duplicate with pooled controls of colostrum. Colostrum protein was analyzed on a single plate, with an intraassay CV of 1.4%. The ­intraassay and interassay CV were 3.0% and 8.6% for triglycerides, 2.3% and 3.9% for lactose, 2.0% and 7.2% for free glucose, and 2.8% and 1.4% for urea N, respectively.

Colostral immunoglobulin (**Ig**) G and A isoforms were analyzed by a colorimetric sandwich enzyme linked immunosorbent assay (**ELISA**) using Bovine IgG and IgA ELISA Kits (Bethyl Laboratories, Inc., Montgomery, TX) with pre-coated plate strips, following the manufacturer’s instructions. Colostrum samples were thawed at 4°C and diluted 1:1,000,000 for IgG and 1:50,000 for IgA in sterile polypropylene tubes using the kit-provided dilution buffer. Standards, controls, and samples were plated in duplicate with the standard curve placed in the middle, with samples and a pooled control on either side. Plates were read at 450 nm on a UV-visible microplate reader (Biotek Synergy HT, Biotek Instruments Inc., Winooski, VT). Sample concentrations were calculated using the four-parameter non-linear standard curve and corrected for dilution. The intraassay and interassay CV for colostrum IgG were 4.9% and 7.5%, respectively. Colostrum IgA was analyzed on a single plate, and the intraassay CV was 2.2%.

### Statistical analyses

One heifer (CON) was removed from the study completely due to late gestational abortion, resulting in CON *n* = 12 and NR *n* = 13. One female (CON) was observed being suckled by another dam’s calf before calving and thus not included in colostrum yield and composition analyses. Nutrient intakes, maternal performance, gestation length, calf size at birth, and colostrum yield and composition were analyzed using the MIXED procedure in SAS 9.4 (SAS Institute Inc., Cary, NC) with late gestational nutritional plane as a fixed effect and animal as the experimental unit. For gestational circulating metabolites over time, late gestational nutritional plane, day of gestation, and their interaction were considered fixed effects. These were considered repeated measures using the majority best-fit covariance structure (based on Akaike Information Criterion, Bayesian Information Criterion, and corrected Bayesian Information Criterion) specific for each variable (chosen from unstructured, compound symmetry, heterogeneous compound symmetry, autoregressive, and heterogeneous autoregressive). Circulating metabolites at 1 h post-calving were analyzed separately with late gestational nutritional plane as a fixed effect. For all measures except nutrient intakes, Julian date of treatment initiation (to remove variation in breeding date and age of dam) and calf sex (if *P* < 0.25) were included as covariates (fixed effects). PROC TTEST was used to test if average daily gain and change in BCS and backfat thickness were different than 0 within nutritional planes. The proportion of births that required assistance at calving or were abnormal presentations were evaluated by the two-sided Fisher’s exact test using the FREQ procedure of SAS. Means were separated using least significant difference and considered different when *P* ≤ 0.05 and tendencies considered when 0.05 < *P* ≤ 0.10. In the absence of interactions, main effects are discussed.

## Results

### Nutrient intake during gestation

**Figure 1.**
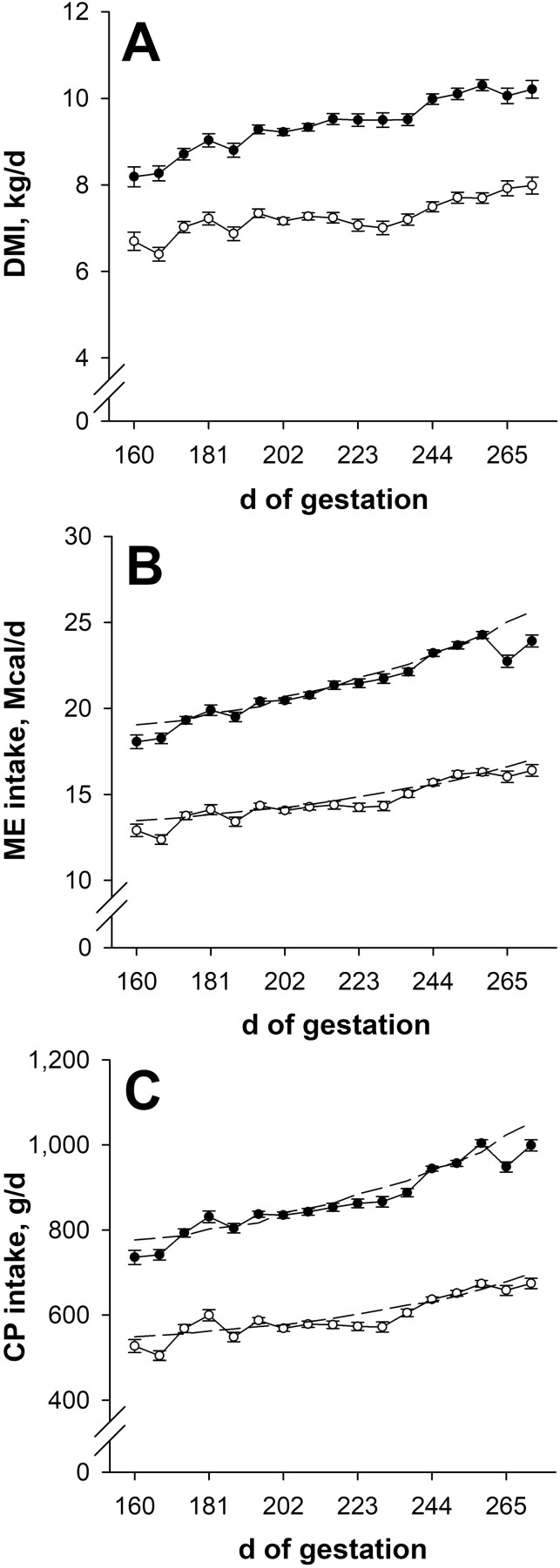
Dry matter intake (DMI; panel A), metabolizable energy intake (ME; panel B), and crude protein intake (CP; panel C) from day 160 of gestation to parturition. Solid circles (● ) represent primiparous beef females individually-fed 100% (Control; *n* = 12) and open circles (◯) represent primiparous beef females individually-fed 70% (Nutrient Restricted; *n* = 13) of metabolizable energy and metabolizable protein requirements for maintenance, pregnancy, and growth from day 160 of gestation to parturition. Least squares means ± SEM are presented. Nutritional plane means differ (*P* < 0.001) on all days for each measure. Targeted weekly ME and CP intakes are represented by the dashed lines.

Dry matter intake, ME intake, and CP intake were less (*P* < 0.001; Figure 1A, B, and C) for NR dams compared with CON during late gestation. Weekly ME and CP intakes of NR dams averaged 98.1% (weekly range: 91.2% to 102.0%) of their ME ­targets and 98.5% (weekly range: 91.4% to 106.6%) of their CP targets during late gestation. For CON dams, weekly ME and CP intakes during late gestation averaged 98.1% (weekly range: 90.8% to 101.5%) and 98.5% (weekly range: 92.6% to 103.6%) of their ME and CP targets, respectively. Weekly DMI during late gestation averaged 22.6% less (*P* < 0.001; range: 18.2% to 26.2%) for NR dams compared with CON. Hay DMI was not affected (*P* ≥ 0.20; data not shown) by nutritional plane for the weeks beginning on days 160 to 265 of gestation; however, NR dams tended to consume more (*P* = 0.10) hay DMI during the week that began on day 272 of gestation.

### Maternal gestational performance and circulating metabolites

**Figure 2.**
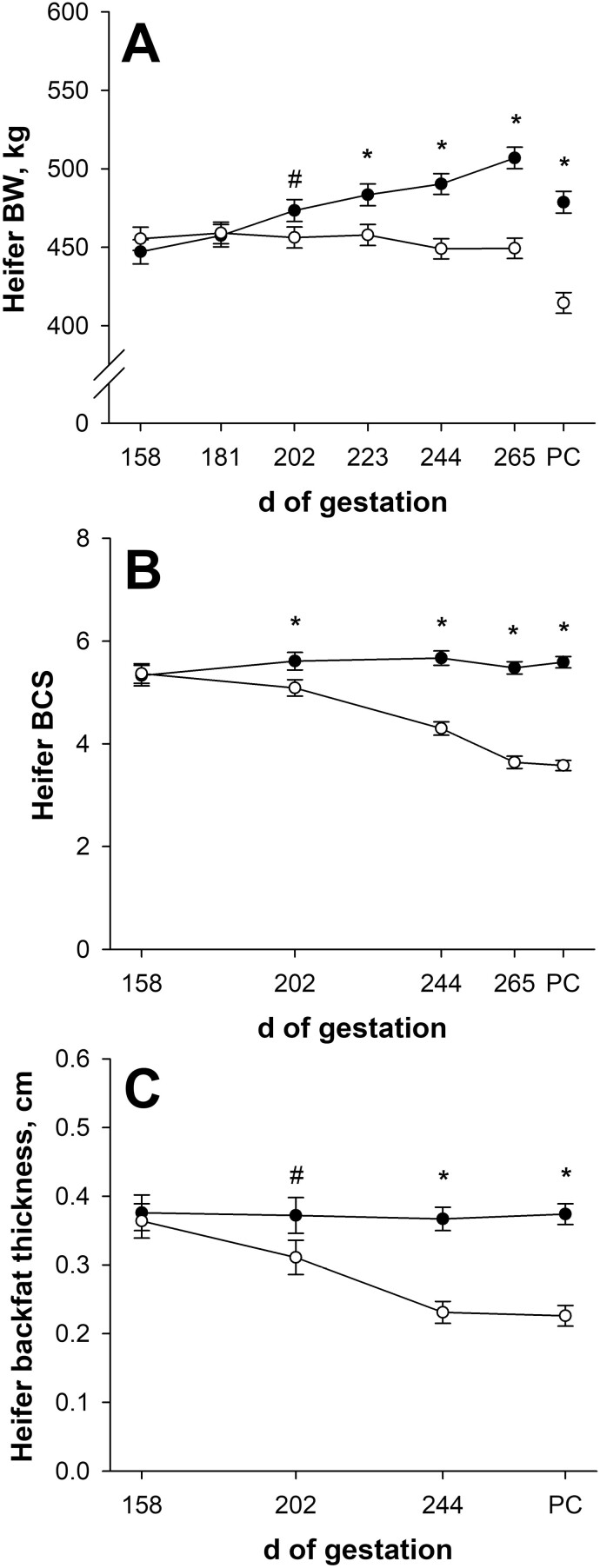
Effects of late gestational nutritional plane on heifer body weight (BW; panel A), body condition score (BCS; panel B), and backfat thickness (panel C) from day 158 of gestation until day 1 post-calving (PC). Solid circles (● ) represent primiparous beef females individually-fed 100% (Control; *n* = 12) and open circles (◯) represent primiparous beef females individually-fed 70% (Nutrient Restricted; *n* = 13) of metabolizable energy and metabolizable protein requirements for maintenance, pregnancy, and growth from day 160 of gestation to parturition. Dam body condition score was assessed on a 1 to 9 scale (1 = emaciated, 9 = obese). Least squares means ± SEM are presented. *Nutritional plane means differ (*P* ≤ 0.05). #Nutritional plane means tend to differ (0.05 < *P* ≤ 0.10).

Initial heifer BW, BCS, and backfat thickness did not differ (*P* ≥ 0.45; Figure 2A, B, and C) between nutritional planes. On day 181 of gestation, pregnant heifer BW did not differ (*P* = 0.87; Figure 2A) between nutritional planes, but by day 202 of gestation, NR dams tended to weigh less (*P* = 0.09) than CON dams. Pregnant heifer BW was less (*P* ≤ 0.01) in NR dams for the remainder of gestation, where NR dams weighed 11.4% less (*P* < 0.001; 449 vs. 507 ± 7 kg) than CON dams on day 265 of gestation. From treatment initiation until day 265 of gestation, pregnant heifer average daily gain (gravid weight) was greater (*P* < 0.001; Table 1) in CON dams compared with NR. Nutrient restriction resulted in pregnant heifer average daily gain that was not different (*P* = 0.15) than 0. Maternal post-calving BW was 13.3% less (*P* < 0.001; 415 vs. 478 ± 7 kg) in NR dams compared with CON. Maternal average daily gain (non-gravid) from day 158 of gestation until post-calving was affected (*P* < 0.001; Table 1) by nutritional plane, where CON dams gained (*P* < 0.001) maternal BW during late gestation while NR dams decreased (*P* < 0.001) maternal BW.

**Table 1.** Effects of late gestational nutritional plane of primiparous beef females on maternal growth and body composition changes during gestation

	Nutritional plane^1^			
Item	Control	Nutrient Restricted	SEM^2^	*P*-value
Average daily gain (gravid), kg/d				
Days 158 to 265 of gestation	0.56	−0.06	0.04	<0.001
Maternal average daily gain (non-gravid), kg/d				
Day 158 of gestation to post-calving^3^	0.35	−0.25	0.03	<0.001
Body condition score change^4^				
Day 158 of gestation to post-calving	0.24	−1.80	0.15	<0.001
Backfat thickness change, cm				
Day 158 of gestation to post-calving	−0.002	−0.138	0.021	<0.001

^1^Primiparous dams were individually-fed either 100% (Control) or 70% (Nutrient Restricted) of metabolizable energy and metabolizable protein requirements for maintenance, pregnancy, and growth from day 160 of gestation to parturition.

^2^Standard error of the mean for Control (*n* = 12) and Nutrient Restricted (*n* = 13).

^3^Non-gravid body weight on day 158 of gestation determined by subtracting the gravid uterine weight.

^4^1 to 9 scale (1 = emaciated, 9 = obese).

On day 202 of gestation, NR dams had less (*P* = 0.04; Figure 2B) BCS and tended to have less (*P* = 0.10; Figure 2C) backfat thickness than CON dams. Body condition score differences remained (*P* < 0.001) throughout gestation, where NR dams had 33.6% less (*P* < 0.001; 3.64 vs. 5.48 ± 0.12) BCS on day 265 of gestation compared with CON dams. On day 244 of gestation, maternal backfat thickness was 37.1% less (*P* < 0.001; 0.231 vs. 0.367 ± 0.017 cm) for NR dams compared with CON dams. Post-calving maternal BCS was 36.0% less (*P* < 0.001; 3.58 vs. 5.59 ± 0.11) and maternal backfat thickness was 39.6% less (*P* < 0.001; 0.226 vs. 0.374 ± 0.015 cm) for NR dams than CON. Body condition score change and backfat thickness change were affected (*P* < 0.001; Table 1) by nutritional plane. During late gestation, CON dam BCS change and backfat thickness change were not different (*P* ≥ 0.17) than 0, but NR dams decreased (*P* < 0.001) in BCS and backfat thickness.

**Figure 3.**
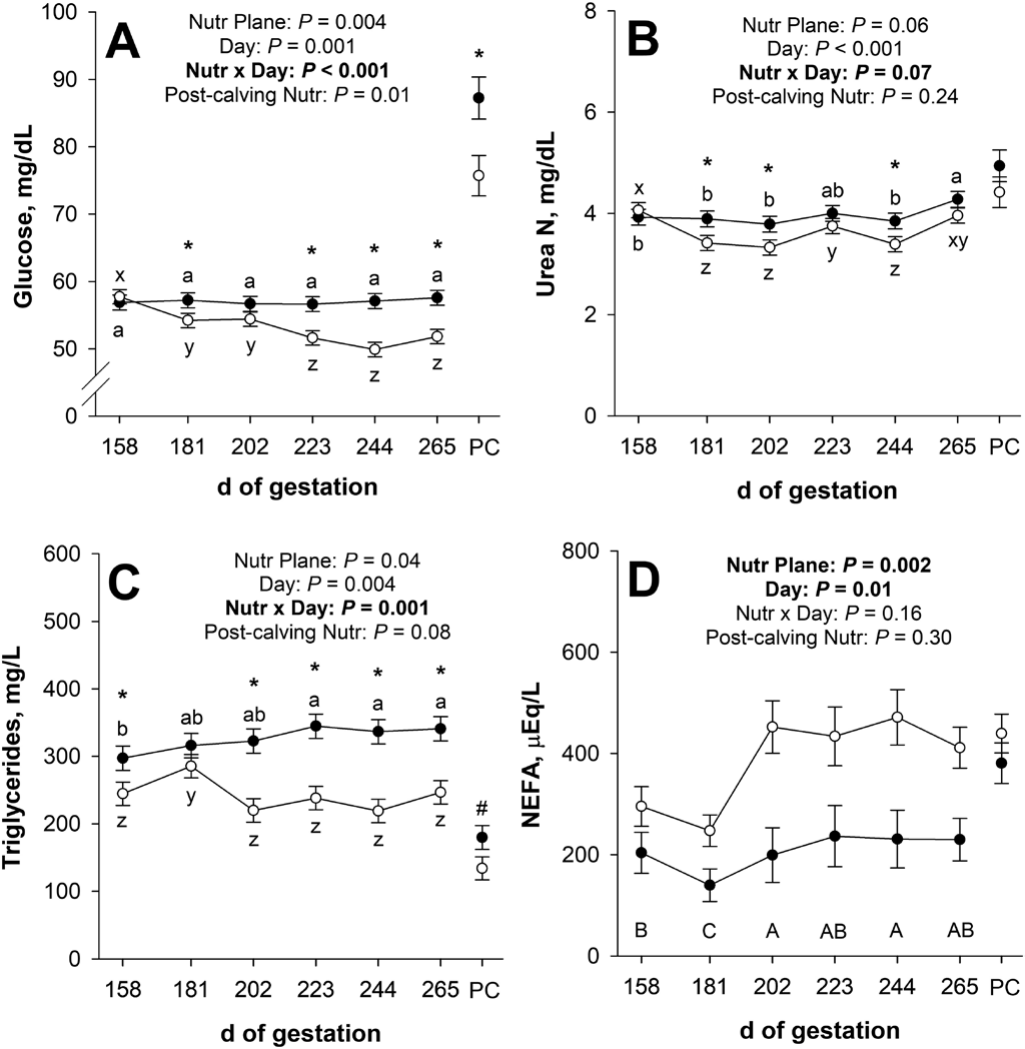
Effects of late gestational nutritional plane on maternal plasma glucose (panel A), serum urea N (panel B), plasma triglycerides (panel C), and serum non-esterified fatty acids (NEFA; panel D) from day 158 of gestation until parturition. Solid circles (● ) represent primiparous beef females individually-fed 100% (Control; *n* = 12) and open circles (◯) represent primiparous beef females individually-fed 70% (Nutrient Restricted; *n* = 13) of metabolizable energy and metabolizable protein requirements for maintenance, pregnancy, and growth from day 160 of gestation to parturition. Least squares means ± SEM are presented. Gestational circulating metabolites were considered repeated measures, 1 h post-calving (PC) circulating metabolites were analyzed separately. *Nutritional plane means within day differ (*P* ≤ 0.05). #Nutritional plane means within day tend to differ (0.05 < *P* ≤ 0.10). ^a,b^Means differ (*P* ≤ 0.05) for Control across days. ^x,y,z^Means differ (*P* ≤ 0.05) for Nutrient Restricted across days. ^A,B,C^Means for main effect of day differ (*P* ≤ 0.05).

There was a nutritional plane × day interaction (*P* < 0.001; Figure 3A) for maternal plasma glucose concentration during late gestation. Initial plasma glucose did not differ (*P* = 0.58) between nutritional planes. Plasma glucose was less (*P* ≤ 0.05) in NR dams than in CON on day 181 of gestation and from day 223 until 265 of gestation. In CON dams, plasma glucose did not change (*P* ≥ 0.65) throughout gestation; however, in NR dams, plasma glucose decreased (*P* ≤ 0.01) from day 158 to 181 and from day 202 to 223 of gestation but tended to increase (*P* = 0.08) from day 244 to 265 of gestation. Plasma glucose at 1 h post-calving was less (*P* = 0.01) for NR dams compared with CON.

There tended to be a nutritional plane × day interaction (*P* = 0.07; Figure 3B) for maternal serum urea N concentration during late gestation. Initial serum urea N did not differ (*P* = 0.53) between nutritional planes. Serum urea N was less (*P* ≤ 0.04) in NR dams compared with CON on days 181, 202, and 244 of gestation, but was not affected (*P* ≥ 0.14) by nutritional plane on days 223 or 265 of gestation. In CON dams, serum urea N did not change (*P* ≥ 0.20) from day 158 to 244 of gestation but increased (*P* = 0.01) from day 244 to 265 of gestation. Serum urea N showed more fluctuation in NR dams, decreasing (*P* < 0.001) from day 158 to 181 of gestation, increasing (*P* = 0.009) from day 202 to 223, decreasing (*P* = 0.03) from day 223 to 244, and increasing (*P* < 0.001) from day 244 to 265 of gestation. At 1 h post-calving, serum urea N was not affected (*P* = 0.24) by late gestational nutritional plane.

Maternal plasma triglyceride concentration was affected (*P* = 0.001; Figure 3C) by the nutritional plane × day interaction during late gestation. Initial plasma triglycerides were 17.7% less (*P* = 0.04) in NR dams compared with CON. Plasma triglycerides were not affected (*P* = 0.22) by nutritional plane on day 181 of gestation, but were 27.7% to 34.9% less (*P* < 0.001) in NR dams from days 202 until 265 of gestation compared with CON dams. In CON dams, plasma triglycerides were greater (*P* ≤ 0.02) from days 223 to 265 of gestation than on day 158 of gestation. In NR dams, plasma triglycerides increased (*P* = 0.01) from day 158 to 181 of gestation, decreased (*P* < 0.001) from day 181 to 202 of gestation, and tended to increase (*P* = 0.09) from day 244 to 265 of gestation. Plasma triglycerides tended to be less (*P* = 0.08) in NR dams compared with CON at 1 h post-calving.

Maternal serum NEFA concentration was not affected (*P* = 0.16; Figure 3D) by the nutritional plane × day interaction during late gestation. There was a main effect of nutritional plane, where NR dams had greater (*P* = 0.002) serum NEFA during late gestation, largely driven by the magnitude of differences between NR and CON dams from days 202 to 265 of gestation. Additionally, there was an effect (*P* = 0.01) of day of gestation on serum NEFA. Serum NEFA decreased (*P* = 0.05) from day 158 to 181 of gestation, increased (*P* < 0.001) from day 181 to 202 of gestation, but did not change (*P* ≥ 0.19) from day 202 to 265 of gestation. At 1 h post-calving, serum NEFA were not affected (*P* = 0.30) by nutritional plane.

### Calving characteristics and calf size at birth

Gestation length was not affected (*P* = 0.46; Table 2; range: 274 to 286 d) by late gestational nutritional plane. The percentage of abnormal presentations was not affected (*P* = 0.22) by nutritional plane, but 3 of 13 calves born to NR dams were abnormally presented while 0 of 12 calves born to CON dams were abnormally presented. An additional dam in each nutritional plane had a manually-assisted delivery, and 1 CON dam had a mechanically-assisted delivery. In total, 4 of 13 NR dams and 2 of 12 CON dams required assistance at calving, but the percentage assisted at calving was not affected (*P* = 0.64) by nutritional plane.

**Table 2.** Effects of late gestational nutritional plane of primiparous beef females on calf size at birth

	Nutritional plane^1^			
Item	Control	Nutrient Restricted	SEM^2^	*P*-value
Gestation length, d	278	279	1	0.46
Calf sex, % male	66.7	69.2	–	–
Assisted calving, %	16.7	30.8	–	0.64
Abnormal presentation, %	0.0	23.1	–	0.22
Calf size^3^				
Birth weight, kg	30.7	29.9	1.5	0.72
Shoulder to rump length, cm	55.4	55.0	0.9	0.76
Heart girth, cm	70.2	69.1	1.1	0.46
Abdominal girth, cm	63.3	62.5	1.2	0.64
Flank girth, cm	58.2	56.9	1.3	0.48
Cannon circumference, cm	11.5	11.3	0.2	0.49
Cannon length, cm	17.2	17.7	0.3	0.27
Coronet circumference, cm	17.7	17.4	0.3	0.35
Height at shoulder, cm	67.0	66.6	0.7	0.68
Calf ponderal index^4^, kg/m^3^	180	180	6	0.99
Heart girth:length^5^	1.27	1.26	0.01	0.50
Volume^6^, L	17.9	17.2	0.9	0.57
Longissimus muscle area^7^, cm^2^	10.8	11.2	0.7	0.65
Birth weight^8^, % initial dam body weight	6.88	6.58	0.32	0.52
Birth weight^9^, % final dam body weight	6.43	7.23	0.33	0.09

^1^Primiparous dams were individually-fed either 100% (Control) or 70% (Nutrient Restricted) of metabolizable energy and metabolizable protein requirements for maintenance, pregnancy, and growth from day 160 of gestation to parturition.

^2^Standard error of the mean for Control (*n* = 12) and Nutrient Restricted (*n* = 13).

^3^Calves were weighed and measured at 0.9 ± 0.3 h (SD) of age (pre-suckling).

^4^Ponderal index = calf birth weight (kg)/shoulder to rump length (m)^3^.

^5^Ratio of heart girth (cm):shoulder to rump length (cm).

^6^Volume (L) = [π × average girth radius (cm)^2^ × shoulder to rump length (cm)]/1,000.

^7^Determined at 7 d of age.

^8^Dam body weight before dietary treatment initiation (days 158 and 159 of gestation).

^9^Dam body weight post-calving (days 1 and 2 of lactation).

Calf birth weight, body size measures, indicators of calf shape, and longissimus muscle area were not affected (*P* ≥ 0.27; Table 2) by late gestational nutritional plane. Calf birth weight as a percent of dam BW on day 158 of gestation was not affected (*P* = 0.52) by late gestational nutritional plane; however, calf birth weight as a percent of dam post-calving BW tended to be greater (*P* = 0.09) for calves born to NR dams compared with calves born to CON.

### Colostrum yield and composition

Colostrum weight and volume were 40.3% and 40.2% less (*P =* 0.04; Table 3), respectively, in NR dams compared with CON. Nutrient restriction resulted in colostrum having greater (*P* = 0.01) protein concentration but lower (*P* ≤ 0.03) free glucose and urea N concentrations. Triglyceride and lactose concentrations in colostrum were not affected (*P* ≥ 0.21) by late gestational nutritional plane. Colostrum from NR dams had greater (*P* ≤ 0.04) IgG and IgA concentrations than colostrum from CON dams. Colostrum of NR dams had less (*P* ≤ 0.03) total lactose, total free glucose, and total urea N than colostrum of CON dams. Total protein and total triglycerides in colostrum were not affected (*P* ≥ 0.67) by late gestational nutrient restriction. Late gestational nutrient restriction did not affect (*P* ≥ 0.55) total IgG or total IgA in colostrum.

**Table 3.** Effects of late gestational nutritional plane of primiparous beef females on single-quarter colostrum yield and composition

	Nutritional plane^1^			
Item	Control	Nutrient Restricted	SEM^2^	*P*-value
Colostrum weight, g	510	304	68	0.04
Colostrum volume, mL	471	282	63	0.04
Composition				
Protein, g/dL	15.4	21.0	1.5	0.01
Triglycerides, g/dL	5.83	7.58	1.02	0.22
Lactose, g/dL	2.80	2.56	0.14	0.21
Free glucose, mg/dL	37.1	27.3	3.0	0.03
Urea N, mg/dL	4.18	3.17	0.33	0.03
Immunoglobulin G, mg/mL	172	271	33	0.04
Immunoglobulin A, mg/mL	8.9	15.3	1.6	0.006
Total components^3^				
Protein, g	62.1	57.1	8.5	0.67
Triglycerides, g	22.7	20.7	4.1	0.73
Lactose, g	14.1	7.4	2.0	0.02
Free glucose, mg	188	77	35	0.03
Urea N, mg	21.6	8.6	3.4	0.01
Immunoglobulin G, g	63.2	72.1	10.8	0.55
Immunoglobulin A, g	4.54	3.94	1.22	0.72

^1^Primiparous dams were individually-fed either 100% (Control) or 70% (Nutrient Restricted) of metabolizable energy and metabolizable protein requirements for maintenance, pregnancy, and growth from day 160 of gestation to parturition.

^2^Standard error of the mean for Control (*n* = 11) and Nutrient Restricted (*n*  = 13).

^3^Total components of colostrum were calculated as concentration multiplied by single-quarter colostrum volume.

## Discussion

The reproducing beef female is faced with the challenge of consuming and partitioning nutrients to satisfy multiple physiological processes above maintenance during a successful yearly production cycle. Almost 65% of a mature beef cow’s yearly energy expenditures are used solely for maintenance, but the remaining 35% used for pregnancy and lactation are imperative if successful beef production is to occur (Freetly, 2019). The primiparous female has perhaps the most challenging task, being expected to grow from approximately 60% of her mature size at breeding to roughly 80% by calving at 2 yr of age (NASEM, 2016) while also developing and using her uterus and mammary gland for the first time.

Late gestational beef females are often challenged with limited nutrient availability, which can affect how nutrients are partitioned between maintenance, growth, pregnancy, and lactation (Short and Adams, 1988). While many past studies have characterized the effects of gestational undernutrition on some of the response variables measured here, the current experiment evaluated the partitioning of nutrients to the dam, fetus, and colostrum simultaneously in the first-parity beef female. The crucial nutrients partitioned to colostrum are presumably accounted for in the physiological function of pregnancy in the list above, but characterization in beef cattle developmental programming research is limited. Overall, our data suggest that late gestational nutrient restriction in heifers slowed maternal growth and instead mobilized maternal body stores. In doing so, nutrients partitioned to fetal growth were spared; however, these specific results do not give any indication of the effect on fetal development. Colostrum yield and total colostral lactose were decreased, but otherwise colostrum total nutrients and Ig available to the newborn calf were not diminished.

Gestational diets were based in poor quality forage fed ad libitum to resemble a production-relevant scenario during late gestation for many cow–calf operations. Hay consumption was not affected by nutrient restriction, and while total DMI was reduced, the reduction never exceeded 2.5 kg DM at any point during gestation. Individual feeding allowed us to precisely feed each female to her estimated individual nutrient requirements and collect nutrient intake data. When combined with the performance responses observed in this and forthcoming papers, these data should allow for improvement of pregnant beef heifer nutrient requirements going forward.

### Nutrient partitioning during late gestation

It has been hypothesized that the first two-thirds of gestation represent the anabolic phase of pregnancy, with the last third serving as the catabolic portion (Naismith and Morgan, 1976; Robinson, 1986). In the current study, heifers were nutrient restricted during late gestation, when the dam is thought to shift towards catabolism if necessary to meet the increased requirements of late pregnancy. Control females gained maternal BW as expected, maintained BCS and backfat thickness, and were metabolically stable during late gestation. Ruminants respond to periods of undernutrition with short-, medium-, and long-term adaptations, altering BW and body composition according to the duration and severity of nutrient restriction (Chilliard et al., 1998). Nutrient restriction altered metabolic status by 21 d of restriction (day 181 of gestation), and heifer BW divergence, loss of BCS and backfat thickness, and elevated NEFA from adipose mobilization were apparent by 42 d of restriction (day 202 of gestation). Post-calving, non-gravid BW of nutrient restricted females was 31.7 kg less and BCS was 1.8 less than at treatment initiation.

As expected, control females gained in both gravid and non-gravid BW across late gestation while maintaining BCS and backfat thickness. During late gestation, control females achieved the maternal growth that was projected based on the ME and MP allocated for gain. A cow does not reach her mature BW until approximately 6 yr of age, but most of the rapid maternal growth occurs before and during the first pregnancy and lactation (Freetly et al., 2021). With the expectation that primiparous females should reach 80% of their mature BW by first calving at 2 yr of age (NASEM, 2016), control females post-calving were at an adequate weight relative to their anticipated mature size (590 kg).

Circulating metabolites in control females were generally stable during late gestation, and their metabolic status indicated that the individual feeding was likely adequate for their gestational requirements. Serum urea N concentrations for control females were less than anticipated, as they were below the threshold of 7 mg/dL that Hammond (1997) suggested indicates protein deficiency, despite maternal performance not being hindered. Circulating urea N is indicative of the ratio of dietary CP to ruminally fermentable organic matter (Oltner et al., 1985), so perhaps targeting both energy and protein needs precisely allowed ample carbon skeleton availability for ruminal microbial transamination and protein synthesis, thus minimizing urea in circulation.

During late gestation, nutrient restricted females lost BCS and backfat thickness, and their post-calving BW and maternal average daily gain reflect that they lost weight at a time when primiparous females are expected to continue growing. As a result, nutrient restricted females were 63.6 kg BW and 2.0 BCS less than control females post-calving. The weight change per unit BCS change between nutritional planes agree with data in primiparous lactating beef heifers (Lalman et al., 1997) and that adopted by NASEM (2016). Maternal BCS and backfat thickness losses in nutrient restricted females followed a similar pattern, and the percent decrease from controls were in good agreement post-calving (36.0% and 39.6%, respectively).

Ruminants respond to periods of undernutrition with a variety of metabolic adaptations in an attempt to decrease their basal metabolic rate and ensure nutrient availability similar to nutrient needs (Chilliard et al., 1998). Some of these metabolic adaptations include the use of glycogen stores, change in passage rate, decreases in organ mass, and mobilization of body tissue stores. Burrin et al. (1990) demonstrated that gastrointestinal organs and liver were smaller after 21 d of nutrient restriction in growing lambs; thus, divergence in BW in our study on day 202 of gestation likely was partially explained by changes in organ masses. The small intestine adapts to both stage of pregnancy and nutrient intake during pregnancy (reviewed by Meyer and Caton, 2016), so its changes were likely dynamic during this period. Other visceral organs such as the heart, lungs, and kidneys are dramatically altered by pregnancy and nutrient intake as well (Stock and Metcalfe, 1994; Thornburg et al., 2006). It is reasonable to assume that the complex interactions between growth, pregnancy, and nutrient restriction resulted in alterations to tissue function and mass, which was ultimately reflected in heifer BW. These adaptations may result in improved efficiency of nutrient use after prolonged restriction (Freetly and Nienaber, 1998; Freetly et al., 2008).

Long-term nutrient restriction results in the mobilization of fat, muscle, and bone in the opposite prioritization of which they are deposited during growth (Chilliard et al., 1998). Maternal backfat thickness and BCS differences paired with elevated NEFA indicate that by 42 d of treatments (day 202 of gestation) nutrient restricted females were heavily reliant on mobilizing adipose reserves to meet their energy demands. Maternal energy balance and NEFA are negatively correlated (Erfle et al., 1974), and NEFA were previously elevated in ewes that were nutrient restricted during mid- and late gestation (Meyer et al., 2010; Vonnahme et al., 2013). If undernutrition is sustained for an extended period, NEFA concentration may begin to decrease as fat stores are exhausted (Chilliard et al., 1998). Our data support that, as NEFA concentration was 127.0% greater in nutrient restricted females than controls on day 202 of gestation, and that difference decreased to 78.9% on day 265 of gestation. The possibility that maternal muscle and bone stores were mobilized was not determined in the current study, but deserves future consideration.

Restricting protein and energy intake simultaneously resulted in decreased circulating glucose and urea N by 21 d of nutrient restriction (day 181 of gestation) and triglycerides by 42 d of nutrient restriction (day 202 of gestation). Both energy and protein were restricted to a similar extent in the current study so that one could not compensate for the other and maintain blood glucose. Decreased serum urea N in nutrient restricted females was expected because it is positively correlated with CP intake and indicative of decreased ammonia concentration from microbial and animal deamination of amino acids (Hammond, 1997). Other studies have also demonstrated less circulating glucose (Long et al., 2009; LeMaster et al., 2017; Taylor et al., 2018) and urea N (Mordhorst et al., 2017; Niederecker et al., 2018) after roughly 3 wk of gestational undernutrition. Taylor et al. (2018) also observed decreased maternal triglycerides when measured after 80 d of gestational undernutrition.

Gravid uterine uptake of glucose, amino acids, and O_2_ increases approximately 3- to 5-fold during late gestation to meet the exponentially increasing demands of the developing fetus and uteroplacenta (Reynolds et al., 1986). In the current study, maternal circulating glucose decreased again in nutrient restricted females between days 202 and 223 of gestation. Nutrient restricted females sustained similar fetal growth to control females, so similar gravid uterine glucose needs relative to reduced available gluconeogenic precursors likely caused the decreasing maternal glucose. Likewise, Long et al. (2009) reported that beef cows who were nutrient restricted during early pregnancy and gestated intrauterine growth restricted (**IUGR**) fetuses generally maintained glucose concentration similar to control females during the restriction, but nutrient restricted females who had non-IUGR fetuses with similar fetal growth to controls had less circulating glucose. Additionally, serum urea N concentration increased from days 244 to 265 for females on both nutritional planes. The fetus and uteroplacenta catabolize a large portion of amino acids for energetic needs, with approximately 30% of the amino acid uptake by the uteroplacenta being returned to the maternal circulation as urea (Ferrell et al., 1983). Perhaps the rise in maternal serum urea N is a result of the dramatic uteroplacental amino acid deamination for energetic ­purposes in late gestation.

### Fetal growth

Nutrient restricted females prioritized nutrient delivery to their fetuses over themselves, sacrificing maternal growth to conserve fetal growth similar to control offspring. This is supported by calf birth weight as a percentage of dam BW post-calving being greater for nutrient restricted females. While the uteroplacenta serves as the mediator for nutrient exchange from maternal to fetal circulation, maternal circulating metabolites suggest that less nutrients were available for fetal growth in nutrient restricted dams. Birth weight is the classic response variable to evaluate effects on fetal growth but does not fully depict differences in calf shape. By collecting an array of size measurements representing skeletal size, soft tissue growth (girth), and muscle area in addition to birth weight, we are confident that fetal growth was not affected by nutrient restriction. Still, fetal growth is not necessarily indicative of fetal development, and the effects of late gestational nutrient restriction on neonatal calf vigor, blood chemistry, and hematology from this study will be in a forthcoming paper.

Previous work in late gestational undernutrition in beef cattle has resulted in various calf birth weight outcomes. In a subset of controlled studies that individually-fed females, used heifers bred to a single sire, or both, birth weight was reduced approximately half of the time (Corah et al., 1975; Bellows and Short, 1978; Kroker and Cummins, 1979; Kennedy et al., 2016a) and did not differ the other half of the time (Anthony et al., 1986; Hough et al., 1990; Martin et al., 1997). In more extensive, less controlled experiments, birth weight was also reduced by nutrient restriction in some experiments (Houghton et al., 1990; Niederecker et al., 2018) but did not differ in others (Bellows et al., 1982; Stalker et al., 2006; Martin et al., 2007). While these studies all examined late gestational nutrient restriction, they varied in the extent of restriction (severity, timing, and duration), nutrients restricted (energy, protein, or both), and how nutritional planes were implemented (pasture, group-fed, or individually-fed). Moreover, sufficient details on treatment implementation and animals used were not always fully reported. Birth weight reductions of greater than 12% were accompanied with shorter body lengths (Kroker and Cummins, 1979), but birth weight reductions of 8% did not result in body length or heart girth differences (Kennedy et al., 2019). Bellows et al. (1982) and Anthony et al. (1986) reported no differences in calf size, which followed birth weight.

The 30.7 kg birth weight observed for control females was less than the expected 34 kg; thus, energy and protein requirements allocated for pregnancy during late gestation were overestimated. Maternal BW and body condition results suggest nutrients allocated for maternal growth were adequate, metabolic status was stable during late gestation, and nutrients allocated to pregnancy were overestimated; thus, fetal growth in control females was likely not limited by nutrient intake. There are many possible factors other than gestational nutrition that may influence birth weight. Utilizing single-sired first-parity females that were mated to a single sire and allocated to treatment by fetal sex allowed us to control many of the additional factors affecting birth weight that have confounded nutritional plane effects in other studies. Gestation length, which can also have a major influence on birth weight (Holland and Odde, 1992), was not affected by late gestational nutritional plane in this study. Previous late gestational nutrient restriction literature has reported no effect (Corah et al., 1975; Anthony et al., 1986; Hough et al., 1990; Martin et al., 1997; Kennedy et al., 2016b) or shortened gestation length in nutrient restricted beef females (Tudor, 1972; Kroker and Cummins, 1979).

Gluckman and Liggins (1984) and Ferrell (1991) suggested that fetal growth rarely, if ever, reaches its full genetic potential because of the many constraining factors involved in the maternal environment. Our lab has previously reported that fall-born calves have reduced birth weight compared with spring-born calves (Wichman et al., 2022), likely due to late gestational heat stress. It should be noted that fall-calving females in the current study were housed in partially-covered pens during late gestation, and no signs of heat stress were apparent. With adequate maternal nutrition, fetal growth is generally less in primiparous dams compared with multiparous dams (Holland and Odde, 1992; Duncan et al., 2023), so the use of first-parity females in the current study may have affected our results. First-parity beef females, who are establishing a gravid uterus for the first time, may respond to gestational undernutrition differently than multiparous cows who have had previous successful pregnancies. Despite this, when these same females were predominantly bred to the same sire in their second parity and managed to meet their nutrient requirements in a normal production setting, calf birth weight for the entire group averaged 30.6 kg (unpublished data), which was similar to what we observed in their first parity.

Hammond (1944) postulated that in pregnancy, when fetal and maternal nutrient needs are in competition because nutrient supply is limited, fetal tissues have priority over maternal tissues. In the previously mentioned studies that also reported maternal BW and BCS, maternal performance decreased irrespective of birth weight differences, supporting the hypothesis that maternal tissues are a lesser priority. The decrease in BW and body condition of nutrient restricted females in the current study was equally or more severe than previous ­studies that decreased fetal growth (Corah et al., 1975; Bellows and Short, 1978; Niederecker et al., 2018), indicating that the restriction was clearly severe enough to manifest differences in fetal growth.

### The act of parturition

Dystocia is a major cause of perinatal calf mortality (Bellows et al., 1987). The current study was not designed with the statistical power to detect differences in binomial data for percentage of assisted calving and abnormal presentation, but late gestational nutritional plane did increase the frequency of both. It was previously hypothesized that undernutrition during late gestation may regulate calf birth weight and limit dystocia rates. Research in the area undoubtedly discredited this notion as there was no reduction of dystocia rates even in instances where birth weights were less (Tudor, 1972; Corah et al., 1975; Bellows and Short, 1978; Kroker and Cummins, 1979). In fact, undernutrition resulted in greater dystocia and stillbirth rates (Hodge et al., 1976) and reduced pelvic area growth in primiparous females (Bellows and Short, 1978; Kroker and Cummins, 1979). The three abnormally-presented calves born to nutrient restricted dams in the current study were the same two types of abnormal presentations observed when late gestational nutrient restriction previously increased malpresentations in heifers (Kroker and Cummins, 1979). It has been hypothesized that malpresentations are caused by reduced fetal righting movements or muscle fatigue near term (Fraser, 1977) or poor uterine muscle tone not correcting fetal posture (Dufty, 1973), both which could be exacerbated by nutrient restriction. It is likely that our continuous monitoring around parturition allowed us to identify more instances where abnormal presentations occurred, and intervention was provided more quickly than is typical for U.S. cow–calf production.

A maternal blood sample was collected at 1 h post-calving to evaluate the effect of late gestational nutrient restriction on the metabolic response to parturition. At this time, circulating glucose and triglyceride concentrations were less in nutrient restricted females than controls. Compared with circulating metabolites on day 265 of gestation, parturition appeared to cause a sharp increase in glucose and sharp decrease in triglycerides across both nutritional planes. Serum urea N was minimally affected by parturition, while the serum NEFA of control females increased dramatically. The spike in blood glucose (Anthony et al., 1986) and NEFA (Vazquez-Añon et al., 1994) at parturition has been observed previously as the result of elevated maternal glucocorticoids and catecholamines (Vannucchi et al., 2015). The lack of a dramatic increase in serum NEFA at calving in nutrient restricted females could indicate that adipose reserves were generally exhausted after approximately 120 d of nutrient restriction. Maternal backfat thickness in nutrient restricted females did not decrease from day 244 until calving, further suggesting that maternal adipose mobilization had lessened. Vernon (1992) postulated that this pattern of NEFA was due to the lipolytic response to catecholamines becoming blunted, as a mechanism to prolong survival.

### Colostrum production

Colostrum consumption is critical in the bovine neonate, not just in providing Ig, but also supplying a concentrated source of proteins, lipids, carbohydrates, vitamins, minerals, and many other non-nutritive factors (Blum and Hammon, 2000). The prepartum transfer of colostral Ig and macromolecules from maternal circulation into the mammary gland, or colostrogenesis, occurs in the few months to weeks leading up to parturition (Barrington et al., 2001; McGee et al., 2006). Despite this, the effects of nutrient restriction during late gestation on colostrum production in beef cattle is poorly understood.

In the current study, late gestational nutrient restriction resulted in 40% less colostrum yield in beef heifers. Previously, colostrum yield tended to decrease (Kennedy et al., 2019) or was not affected (Odde, 1988; McGee et al., 2006) by late gestational undernutrition in beef cattle. Nutrient restriction during mid- and late gestation in sheep also resulted in colostrum yield reductions between 33% and 63% (Banchero et al., 2006; Swanson et al., 2008; Meyer et al., 2011).

Passive transfer of immunity from dam to calf occurs solely by ingestion of colostral antibodies because Ig are not transported across the ruminant placenta to the fetus during pregnancy (Larson et al., 1980). The current study suggests that total IgG and IgA transfer was not affected by nutrient restriction, but that decreased colostrum yield caused by reduced lactose production resulted in greater Ig concentration in nutrient restricted dams. These findings agree with previous IgG and IgM results from late gestational protein restriction in beef heifers (Odde, 1988). Other studies in mature beef cows have observed no effect of late gestational undernutrition on Ig concentration, resulting in similar total colostral IgG in Kennedy et al. (2019) but reduced total colostral Ig in McGee et al. (2006). Of these studies, McGee et al. (2006) had the shortest duration of nutrient restriction (last 15 d prepartum) when colostrogenesis was well underway, so it is surprising that total Ig were reduced. Gestational nutrient restriction in sheep resulted in greater IgG concentration, but the reduction in colostrum yield was too severe and total colostral IgG was still reduced (Swanson et al., 2008). Regulation of colostrogenesis includes both endocrine-mediated and Ig transporter-related processes (Barrington et al., 2001), which may have been altered to support similar or enhanced Ig transfer during nutrient restriction.

Total colostrum nutrients available to the neonatal calf are rarely reported in beef cattle. We observed increased colostral protein concentration, decreased free glucose and urea N concentrations, but no change in triglyceride and lactose concentrations due to late gestational nutrient restriction. Nutrient restricted calves had less total lactose available, which is a critical energy source for neonates, but similar total protein and triglycerides from colostrum. In a previous study utilizing mature beef cows, McGee et al. (2006) reported that late gestational nutrient restriction increased fat concentration, but protein and lactose concentrations were not affected. Mid- and late gestational nutrient restriction in ewe lambs has generally reduced total colostral lactose, protein, fat, and urea N (Swanson et al., 2008; Meyer et al., 2011). Previous late gestational nutrient restriction work in multiparous ewes increased colostral protein concentration, decreased lactose concentration, but did not affect fat concentration (Banchero et al., 2006).

Glucose taken up from maternal circulation is an important precursor for lactose synthesis in mammary secretory cells (Hardwick et al., 1961). Furthermore, lactose is the primary osmotic component of mammary secretions ­driving the uptake of water (Linzell and Peaker, 1971); thus, lactose yield and milk yield are strongly correlated (Pollott, 2004). Nutrient restricted females in the current study had less circulating glucose during late gestation and at the time of colostrum collection, which limited the synthesis of lactose for colostrum and resulted in the observed colostrum yield reduction. This is reinforced by the reduction in free glucose also observed in the colostrum of nutrient restricted females. Free glucose cannot be synthesized in the mammary epithelial cells and is representative of reduced glucose absorbed from maternal circulation (Larsen and Moyes, 2015).

Triglycerides were measured in the current study and represent approximately 98% of fat in colostrum (Walstra and Jenness, 1984). The magnitude of increased concentration of colostral triglycerides in nutrient restricted females was greater in the current study than the increased fat concentration observed in McGee et al. (2006), but our triglyceride concentrations were quite variable, and significance was not reached. Greater protein concentration in colostrum from nutrient restricted dams likely reflects greater Ig rather than casein. Urea N consumed in colostrum is not used nutritionally by neonatal beef calves because functional rumen microbes with urease capacity are not established yet (Lengemann and Allen, 1959). Instead, urea N in colostrum is an indicator of protein status of the dam at parturition. In the current study, there was a strong positive correlation (r = 0.85, *P* < 0.001) between colostral urea N and maternal serum urea N determined at the same time post-calving.

We have previously demonstrated that the analytical methods used to determine colostrum nutrients can alter the interpretation (Rathert-Williams et al., 2023), but severity of nutrient restriction, species, and parity differences should also be considered. The profound effects observed in our primiparous beef females and previously in ewe lambs stress the vulnerability of the mammary gland the first time it goes through the process of colostrogenesis. Additionally, how these effects in the first parity may affect long-term mammary function and productivity deserves investigation.

## Conclusions

Primiparous females provided adequate energy and protein during late gestation grew as expected and maintained body condition, but nutrient restricted heifers slowed maternal growth and relied on the mobilization of maternal body stores to meet nutrient demands of late pregnancy. Late gestational nutrient restriction reduced maternal circulating post-absorptive nutrients, but contrary to our hypothesis, the delivery of nutrients to the fetus was not severely altered as fetal growth was spared. Less total colostral lactose production resulted in decreased colostrum yield in nutrient restricted females, but other nutrients and Ig were concentrated so that total amounts available to calves were not affected. Data from the current study indicate that beef heifers experiencing late gestational nutrient restriction prioritized partitioning nutrients to fetal growth and colostrum production over maternal body tissues. These outcomes have not always occurred in previous work examining late gestational nutrient restriction, so continued investigation into how and why nutrients are partitioned across the competing nutrient sinks is warranted.

## Abbreviations:

ADF: acid detergent fiber
BCS: body condition score
BW: body weight
CP: crude protein
DDGS: dried distillers grains with solubles
DM: dry matter
DMI: dry matter intake
ELISA: enzyme linked immunosorbent assay
Ig: immunoglobulin
IUGR: intrauterine growth restriction
ME: metabolizable energy
MP: metabolizable protein
NDF: neutral detergent fiber
NEFA: non-esterified fatty acids
TDN: total digestible nutrients

## References

[CIT0001] Anthony, R. V., R. A. Bellows, R. E. Short, R. B. Staigmiller, C. C. Kaltenbach, and T. G. Dunn. 1986. Fetal growth of beef calves. I. Effect of prepartum dietary crude protein on birth weight, blood metabolites and steroid hormone concentrations. J. Anim. Sci. 62:1363–1374. doi:10.2527/jas1986.6251363x 3722022

[CIT0002] Banchero, G. E., R. P. Clariget, R. Bencini, D. R. Lindsay, J. T. B. Milton, and G. B. Martin. 2006. Endocrine and metabolic factors involved in the effect of nutrition on the production of colostrum in female sheep. Reprod. Nutr. Dev. 46:447–460. doi:10.1051/rnd:2006024 16824452

[CIT0003] Barrington, G. M., T. B. McFadden, M. T. Huyler, and T. E. Besser. 2001. Regulation of colostrogenesis in cattle. Livest. Prod. Sci. 70:95–104. doi:10.1016/S0301-6226(01)00201-9

[CIT0004] Bellows, R. A., D. J. Patterson, P. J. Burfening, and D. A. Phelps. 1987. Occurrence of neonatal and postnatal mortality in range beef cattle. II. Factors contributing to calf death. Theriogenology. 28:573–586. doi:10.1016/0093-691x(87)90274-3 16726340

[CIT0005] Bellows, R. A., and R. E. Short. 1978. Effects of precalving feed level on birth weight, calving difficulty and subsequent fertility. J. Anim. Sci. 46:1522–1528. doi:10.2527/jas1978.4661522x

[CIT0006] Bellows, R. A., R. E. Short, and G. V. Richardson. 1982. Effects of sire, age of dam and gestation feed level on dystocia and postpartum reproduction. J. Anim. Sci. 55:18–27. doi:10.2527/jas1982.55118x

[CIT0007] Blum, J. W., and H. Hammon. 2000. Colostrum effects on the gastrointestinal tract, and on nutritional, endocrine and metabolic parameters in neonatal calves. Livest. Prod. Sci. 66:151–159. doi:10.1016/S0301-6226(00)00222-0

[CIT0008] Burrin, D. G., C. L. Ferrell, R. A. Britton, and M. Bauer. 1990. Level of nutrition and visceral organ size and metabolic activity in sheep. Br. J. Nutr. 64:439–448. doi:10.1079/bjn19900044 2223745

[CIT0009] Caton, J. S., and B. W. Hess. 2010. Maternal plane of nutrition: impacts on fetal outcomes and postnatal offspring responses. In: Proc. 4th Grazing Livestock Nutrition Conference. West. Sect. Am. Soc. Anim. Sci., Champaign, IL; p. 104–122.

[CIT0010] Chilliard, Y., F. Bocquier, and M. Doreau. 1998. Digestive and metabolic adaptations of ruminants to undernutrition, and consequences on reproduction. Reprod. Nutr. Dev. 38:131–152. doi:10.1051/rnd:19980201 9638788

[CIT0011] Corah, L. R., T. G. Dunn, and C. C. Kaltenbach. 1975. Influence of prepartum nutrition on the reproductive performance of beef females and the performance of their progeny. J. Anim. Sci. 41:819–824. doi:10.2527/jas1975.413819x 1158813

[CIT0012] DelCurto, T., B. W. Hess, J. E. Huston, and K. C. Olson. 2000. Optimum supplementation strategies for beef cattle consuming low-quality roughages in the western United States. J. Anim. Sci. 77:1–16. doi:10.2527/jas2000.77e-suppl1v

[CIT0013] Dufty, J. H. 1973. Clinical studies on bovine parturition—foetal aspects. Aust. Vet. J. 49:177–182. doi:10.1111/j.1751-0813.1973.tb06781.x 4711143

[CIT0014] Duncan, N. B., K. S. Stoecklein, A. P. Foote, and A. M. Meyer. 2023. Dam parity affects fetal growth, placental size, and neonatal metabolism in spring-born beef calves. J. Anim. Sci. 101:skac399. doi:10.1093/jas/skac399 36478071 PMC9883719

[CIT0015] Erfle, J. D., L. J. Fisher, and F. D. Sauer. 1974. Interrelationships between blood metabolites and an evaluation of their use as criteria of energy status of cows in early lactation. Can. J. Anim. Sci. 54:293–303. doi:10.4141/cjas74-041

[CIT0016] Ferrell, C. L. 1991. Maternal and fetal influences on uterine and conceptus development in the cow: I. Growth of tissues of the gravid uterus. J. Anim. Sci. 69:1945–1953. doi:10.2527/1991.6951945x 1712353

[CIT0017] Ferrell, C. L., S. P. Ford, R. L. Prior, and R. K. Christenson. 1983. Blood flow, steroid secretion and nutrient uptake of the gravid bovine uterus and fetus. J. Anim. Sci. 56:656–667. doi:10.2527/jas1983.563656x 6841301

[CIT0018] Ferrell, C. L., W. N. Garrett, and N. Hinman. 1976. Growth, development and composition of the udder and gravid uterus of beef heifers during pregnancy. J. Anim. Sci. 42:1477–1489. doi:10.2527/jas1976.4261477x 931823

[CIT0019] Fraser, A. F. 1977. Fetal kinesis and a condition of fetal inertia in equine and bovine subjects. Appl. Anim. Ethol. 3:89–90. doi:10.1016/0304-3762(77)90074-8

[CIT0020] Freetly, H. C. 2019. Fiftieth Anniversary of the California Net Energy System Symposium: what are the energy coefficients for cows? Transl. Anim. Sci. 3:969–975. doi:10.1093/tas/txz024 32704861 PMC7200495

[CIT0021] Freetly, H. C., R. A. Cushman, and G. L. Bennett. 2021. Production performance of cows raised with different postweaning growth patterns. Transl. Anim. Sci. 5:1–7. doi:10.1093/tas/txab031 PMC8262759 34250449

[CIT0082] Freetly, H. C., C. L. Ferrell, and T. G. Jenkins. 2005. Nutritionally altering weight gain patterns of pregnant heifers and young cows changes the time that feed resources are offered without any differences in production. J. Anim. Sci. 83:916–926. doi:10.2527/2005.834916x 15753348

[CIT0023] Freetly, H. C., and J. A. Nienaber. 1998. Efficiency of energy and nitrogen loss and gain in mature cows. J. Anim. Sci. 76:896–905. doi:10.2527/1998.763896x 9535353

[CIT0024] Freetly, H. C., J. A. Nienaber, and T. Brown-Brandl. 2008. Partitioning of energy in pregnant beef cows during nutritionally induced body weight fluctuation. J. Anim. Sci. 86:370–377. doi:10.2527/jas.2007-0250 17998430

[CIT0025] Gluckman, P. D., and G. C. Liggins. 1984. Regulation of fetal growth. In: Beard, R. W. and P. W. Nathanielsz, editors. Fetal physiology and medicine. 2nd rev. edn. New York, NY: Butterworths; p. 511–557.

[CIT0026] Hammond, J. 1944. Physiological factors affecting birth weight. Proc. Nutr. Soc. 2:8–14.

[CIT0027] Hammond, A. C. 1997. Update on bun and mun as a guide for protein supplementation in cattle. In: 8th Annual Florida Ruminant Nutrition Symposium; Gainesville, FL: University of Florida; p. 43–52.

[CIT0028] Hardwick, D. C., J. L. Linzell, and S. M. Price. 1961. The effect of glucose and acetate on milk secretion by the perfused goat udder. Biochem. J. 80:37–45. doi:10.1042/bj0800037 13711505 PMC1243948

[CIT0029] Hodge, P. B., R. C. Beasley, and J. Stokoe. 1976. Effect of three levels of grazing nutrition upon calving and subsequent performance in Hereford heifers. Proc. Aust. Soc. Anim. Prod. 11:245–248.

[CIT0030] Holland, M. D., and K. G. Odde. 1992. Factors affecting calf birth weight: a review. Theriogenology. 38:769–798. doi:10.1016/0093-691x(92)90155-k 16727179

[CIT0031] Holman, J., Y. Assefa, M. Stamm, and A. K. Obour. 2021. Canola yield, forage accumulation, and nutritive value in dual-purpose and companion cropping. Crop Sci. 61:814–824. doi:10.1002/csc2.20291

[CIT0032] Hough, R. L., F. D. McCarthy, H. D. Kent, D. E. Eversole, and M. L. Wahlberg. 1990. Influence of nutritional restriction during late gestation on production measures and passive immunity in beef cattle. J. Anim. Sci. 68:2622–2627. doi:10.2527/1990.6892622x 2211390

[CIT0033] Houghton, P. L., R. P. Lemenager, L. A. Horstman, K. S. Hendrix, and G. E. Moss. 1990. Effects of body composition, pre-and postpartum energy level and early weaning on reproductive performance of beef cows and preweaning calf gain. J. Anim. Sci. 68:1438–1446. doi:10.2527/1990.6851438x 2365654

[CIT0034] Kennedy, V. C., M. L. Bauer, K. C. Swanson, and K. A. Vonnahme. 2016a. Supplementation of corn dried distillers grains plus solubles to gestating beef cows fed low-quality forage: I. Altered intake behavior, body condition, and reproduction. J. Anim. Sci. 94:240–247. doi:10.2527/jas.2015-9615 26812330

[CIT0035] Kennedy, V. C., J. J. Gaspers, B. R. Mordhorst, G. L. Stokka, K. C. Swanson, M. L. Bauer, and K. A. Vonnahme. 2019. Late gestation supplementation of corn dried distiller’s grains plus solubles to beef cows fed a low-quality forage: III. Effects on mammary gland blood flow, colostrum and milk production, and calf body weights. J. Anim. Sci. 97:3337–3347. doi:10.1093/jas/skz201 31181138 PMC6667239

[CIT0036] Kennedy, V. C., B. R. Mordhorst, J. J. Gaspers, M. L. Bauer, K. C. Swanson, C. O. Lemley, and K. A. Vonnahme. 2016b. Supplementation of corn dried distillers’ grains plus solubles to gestating beef cows fed low-quality forage: II. Impacts on uterine blood flow, circulating estradiol-17β and progesterone, and hepatic steroid metabolizing enzyme activity. J. Anim. Sci. 94:4619–4628. doi:10.2527/jas.2016-0400 27898957

[CIT0037] Kroker, G. A., and L. J. Cummins. 1979. The effect of nutritional restriction on Hereford heifers in late pregnancy. Aust. Vet. J. 55:467–474. doi:10.1111/j.1751-0813.1979.tb00371.x 539931

[CIT0038] Lalman, D. L., D. H. Keisler, J. E. Williams, E. J. Scholljegerdes, and D. M. Mallett. 1997. Influence of postpartum weight and body condition change on duration of anestrus by undernourished suckled beef heifers. J. Anim. Sci. 75:2003–2008. doi:10.2527/1997.7582003x 9263044

[CIT0039] Larsen, T., and K. M. Moyes. 2015. Are free glucose and glucose-6-phosphate in milk indicators of specific physiological states in the cow? Animal. 9:86–93. doi:10.1017/S1751731114002043 25159717

[CIT0040] Larson, B. L., H. L. Heary, Jr, and J. E. Devery. 1980. Immunoglobulin production and transport by the mammary gland. J. Dairy Sci. 63:665–671. doi:10.3168/jds.S0022-0302(80)82988-2 7189763

[CIT0041] Larson-Peine, J. M., M. C. Heller, A. R. Rathert-Williams, K. A. Pearl, N. B. Duncan, B. L. Vander Ley, and A. M. Meyer. 2022. Blood chemistry and rectal temperature changes in a population of healthy, fall-born, suckling beef calves from birth to 72 h of age. Theriogenology. 188:145–155. doi:10.1016/j.theriogenology.2022.05.024 35689944

[CIT0042] LeMaster, C. T., R. K. Taylor, R. E. Ricks, and N. M. Long. 2017. The effects of late gestation maternal nutrient restriction with or without protein supplementation on endocrine regulation of newborn and postnatal beef calves. Theriogenology. 87:64–71. doi:10.1016/j.theriogenology.2016.08.004 27613252

[CIT0043] Lengemann, F. W., and N. N. Allen. 1959. Development of rumen function in the dairy calf. II. Effect of diet upon characteristics of the rumen flora and fauna of young calves. J. Dairy Sci. 42:1171–1181. doi:10.3168/jds.S0022-0302(59)90709-X

[CIT0044] Linzell, J. L., and M. Peaker. 1971. Mechanism of milk secretion. Physiol. Rev. 51:564–597. doi:10.1152/physrev.1971.51.3.564 4931980

[CIT0045] Long, N. M., K. A. Vonnahme, B. W. Hess, P. W. Nathanielsz, and S. P. Ford. 2009. Effects of early gestational undernutrition on fetal growth, organ development, and placentomal composition in the bovine. J. Anim. Sci. 87:1950–1959. doi:10.2527/jas.2008-1672 19213703

[CIT0046] Martin, G. S., G. E. Carstens, T. L. Taylor, C. R. Sweatt, A. G. Eli, D. K. Lunt, and S. B. Smith. 1997. Prepartum protein restriction does not alter norepinephrine-induced thermogenesis or brown adipose tissue function in newborn calves. J. Nutr. 127:1929–1937. doi:10.1093/jn/127.10.1929 9311947

[CIT0047] Martin, J. L., K. A. Vonnahme, D. Adams, G. Lardy, and R. N. Funston. 2007. Effects of dam nutrition on growth and reproductive performance of heifer calves. J. Anim. Sci. 85:841–847. doi:10.2527/jas.2006-337 17085735

[CIT0048] McGee, M., M. J. Drennan, and P. J. Caffrey. 2006. Effect of age and nutrient restriction pre partum on beef suckler cow serum immunoglobulin concentrations, colostrum yield, composition and immunoglobulin concentration and immune status of their progeny. Irish J. Agric. Food Res. 45:157–171.

[CIT0049] McGee, M., and B. Earley. 2019. Review: passive immunity in beef-suckler calves. Animal. 13:810–825. doi:10.1017/S1751731118003026 30458893

[CIT0050] Meyer, A. M., and J. S. Caton. 2016. Role of the small intestine in developmental programming: impact of maternal nutrition on the dam and offspring. Adv. Nutr. 7:169–178. doi:10.3945/an.115.010405 27180380 PMC4717893

[CIT0051] Meyer, A. M., J. J. Montonye, J. J. Reed, L. P. Reynolds, D. A. Redmer, J. S. Caton, and K. A. Vonnahme. 2010. Effects of nutritional plane and selenium supply during gestation on circulating non-esterified fatty acid and thyroid hormone concentrations in first-parity ewe lambs. J. Anim. Sci. 88:96.19749015

[CIT0052] Meyer, A. M., J. J. Reed, T. L. Neville, J. F. Thorson, K. R. Maddock-Carlin, J. B. Taylor, L. P. Reynolds, D. A. Redmer, J. S. Luther, C. J. Hammer, et al. 2011. Nutritional plane and selenium supply during gestation affect yield and nutrient composition of colostrum and milk in primiparous ewes. J. Anim. Sci. 89:1627–1639. doi:10.2527/jas.2010-3394 21521822

[CIT0053] Mordhorst, B. R., C. A. Zimprich, L. E. Camacho, M. L. Bauer, and K. A. Vonnahme. 2017. Supplementation of distiller’s grains during late gestation in beef cows consuming low‐quality forage decreases uterine, but not mammary, blood flow. J. Anim. Physiol. Anim. Nutr. 101:e154–e164. doi:10.1111/jpn.12580 27874218

[CIT0054] Naismith, D. J., and B. L. G. Morgan. 1976. The biphasic nature of protein metabolism during pregnancy in the rat. Br. J. Nutr. 36:563–566. doi:10.1079/bjn19760109 1009078

[CIT0055] NASEM. 2016. Nutrient requirements of beef cattle. 8th rev. ed. Washington, DC: Natl. Acad. Press. doi:10.17226/19014

[CIT0056] NFTA. 1993. (National Forage Testing Association) Estimates of energy availability. Appendix A3. https://www.foragetesting.org/lab-procedures. Accessed June 2023.

[CIT0057] Niederecker, K. N., J. M. Larson, R. L. Kallenbach, and A. M. Meyer. 2018. Effects of feeding stockpiled tall fescue versus summer-baled tall fescue-based hay to late gestation beef cows: I. Cow performance, maternal metabolic status, and fetal growth. J. Anim. Sci. 96:4618–4632. doi:10.1093/jas/sky341 30137366 PMC6247859

[CIT0058] NRC. 2000. Nutrient requirements of beef cattle. 7th rev. edn. Washington, DC: Natl. Acad. Press.

[CIT0059] Odde, K. C. 1988. Survival of the neonatal calf. Vet. Clin. North Am. Food Anim. Pract. 4:501–508. doi:10.1016/S0749-0720(15)31027-6 3064888

[CIT0060] Oltner, R., M. Emanuelson, and H. Wiktorsson. 1985. Urea concentrations in milk in relation to milk yield, live weight, lactation number and amount and composition of feed given to dairy cows. Livest. Prod. Sci. 12:47–57. doi:10.1016/0301-6226(85)90039-9

[CIT0061] Pollott, G. E. 2004. Deconstructing milk yield and composition during lactation using biologically based lactation models. J. Dairy Sci. 87:2375–2387. doi:10.3168/jds.S0022-0302(04)73359-7 15328258

[CIT0062] Rathert-Williams, A. R., A. L. Kenny, B. Vardhanabhuti, T. B. McFadden, and A. M. Meyer. 2023. Colorimetric methods for accurate determination of nutrient composition in beef cow colostrum and milk. J. Anim. Sci. 101:skad088. doi:10.1093/jas/skad088 36961880 PMC10119698

[CIT0063] Reynolds, L. P., P. P. Borowicz, J. S. Caton, K. A. Vonnahme, J. S. Luther, C. J. Hammer, K. R. Maddock Carlin, A. T. Grazul-Bilska, and D. A. Redmer. 2010. Developmental programming: the concept, large animal models, and the key role of uteroplacental vascular development. J. Anim. Sci. 88:E61–E72. doi:10.2527/jas.2009-2359 20023136

[CIT0064] Reynolds, L. P., C. L. Ferrell, D. A. Robertson, and S. P. Ford. 1986. Metabolism of the gravid uterus, foetus and utero-placenta at several stages of gestation in cows. J. Agric. Sci. 106:437–444. doi:10.1017/s0021859600063309

[CIT0065] Robinson, J. J. 1986. Changes in body composition during pregnancy and lactation. Proc. Nutr. Soc. 45:71–80. doi:10.1079/pns19860037 3703866

[CIT0066] Short, R. E., and D. C. Adams. 1988. Nutritional and hormonal interrelationships in beef cattle reproduction. Can. J. Anim. Sci. 68:29–39. doi:10.4141/cjas88-003

[CIT0067] Stalker, L. A., D. C. Adams, T. J. Klopfenstein, D. M. Feuz, and R. N. Funston. 2006. Effects of pre-and postpartum nutrition on reproduction in spring calving cows and calf feedlot performance. J. Anim. Sci. 84:2582–2589. doi:10.2527/jas.2005-640 16908664 PMC7109832

[CIT0068] Stock, M. K., and J. Metcalfe. 1994. Maternal physiology during gestation. In: Knobil, E., and J. D. Neill, editors. The physiology of reproduction. 2nd edn. New York, NY: Raven Press; p. 947–983.

[CIT0069] Swanson, T. J., C. J. Hammer, J. S. Luther, D. B. Carlson, J. B. Taylor, D. A. Redmer, T. L. Neville, J. J. Reed, L. P. Reynolds, J. S. Caton, et al. 2008. Effects of gestational plane of nutrition and selenium supplementation on mammary development and colostrum quality in pregnant ewe lambs. J. Anim. Sci. 86:2415–2423. doi:10.2527/jas.2008-0996 18441080

[CIT0070] Taylor, R. K., C. T. LeMaster, K. S. Mangrum, R. E. Ricks, and N. M. Long. 2018. Effects of maternal nutrient restriction during early or mid-gestation without realimentation on maternal physiology and foetal growth and development in beef cattle. Animal. 12:312–321. doi:10.1017/S175173111700163X 28697817

[CIT0071] Thornburg, K. L., S. P. Bagby, and G. D. Giraud. 2006. Maternal adaptation to pregnancy. In: Neill, J. D., T. M. Plant, D. W. Pfaff, J. R. G. Challis, D. M. de Kretser, J. S. Richards, and P. Wassarman, editors. Knobil and Neill’s physiology of reproduction. 3rd edn. St. Louis, MO: Elsevier; p. 2899–2923.

[CIT0072] Tudor, G. D. 1972. Effect of pre-and post-natal nutrition on the growth of beef cattle I. The effect of nutrition and parity of the dam on calf birth weight. Aust. J. Agric. Res. 23:389–395. doi:10.1071/AR9720389

[CIT0073] Van Soest, P. J., J. B. Robertson, and B. A. Lewis. 1991. Methods for dietary fiber, neutral detergent fiber, and nonstarch polysaccharides in relation to animal nutrition. J. Dairy Sci. 74:3583–3597. doi:10.3168/jds.S0022-0302(91)78551-2 1660498

[CIT0074] Vannucchi, C. I., J. A. Rodrigues, L. C. G. Silva, C. F. Lúcio, G. A. L. Veiga, P. V. Furtado, C. A. Oliveira, and M. Nichi. 2015. Association between birth conditions and glucose and cortisol profiles of periparturient dairy cows and neonatal calves. Vet. Rec. 176:358–358. doi:10.1136/vr.102862 25690915

[CIT0075] Vazquez-Añon, M., S. Bertics, M. Luck, R. R. Grummer, and J. Pinheiro. 1994. Peripartum liver triglyceride and plasma metabolites in dairy cows. J. Dairy Sci. 77:1521–1528. doi:10.3168/jds.S0022-0302(94)77092-2 8083410

[CIT0076] Vernon, R. G. 1992. Effects of diet on lipolysis and its regulation. Proc. Nutr. Soc. 51:397–408. doi:10.1079/pns19920053 1480634

[CIT0077] Vonnahme, K. A., T. L. Neville, G. A. Perry, D. A. Redmer, L. P. Reynolds, and J. S. Caton. 2013. Maternal dietary intake alters organ mass and endocrine and metabolic profiles in pregnant ewe lambs. Anim. Reprod. Sci. 141:131–141. doi:10.1016/j.anireprosci.2013.07.010 23981299

[CIT0078] Wagner, J. J., K. S. Lusby, J. W. Oltjen, J. Rakestraw, R. P. Wettemann, and L. E. Walters. 1988. Carcass composition in mature Hereford cows: estimation and effect on daily metabolizable energy requirement during winter. J. Anim. Sci. 66:603–612. doi:10.2527/jas1988.663603x 3378920

[CIT0079] Walstra, P., and R. Jenness. 1984. Lipids. In: Dairy chemistry & physics. New York, NY: John Wiley & Sons; p. 58–97.

[CIT0080] Wichman, L. G., C. A. Redifer, A. R. Rathert-Williams, N. B. Duncan, C. A. Payne, and A. M. Meyer. 2022. Effects of spring-versus fall-calving on perinatal nutrient availability and neonatal vigor in beef cattle. Transl. Anim. Sci. 6:txac136. doi:10.1093/tas/txac136 36381953 PMC9661251

[CIT0081] Wu, G., F. W. Bazer, J. M. Wallace, and T. E. Spencer. 2006. Board-invited review: intrauterine growth retardation: implications for the animal sciences. J. Anim. Sci. 84:2316–2337. doi:10.2527/jas.2006-156 16908634

